# [Retracted] Circular RNA, hsa_circRNA_102049, promotes colorectal cancer cell migration and invasion via binding and suppressing miRNA‑455‑3p

**DOI:** 10.3892/etm.2024.12613

**Published:** 2024-06-18

**Authors:** Yuandong Zhu, Jianjion Li, Haiyuan Liu, Zhengming Song, Qinghua Yang, Chengdong Lu, Wenbin Chen

Exp Ther Med 23:244, 2022; DOI: 10.3892/etm.2022.11169

Following the publication of this paper, it was drawn to the Editor’s attention by a concerned reader that certain of the colony formation assay data shown in Fig. 4C on p. 7 were strikingly similar to data that had already been submitted in different form in another article written by different authors at different research institutes, which has subsequently been retracted: Xia W, Liu Y, Du Y, Cheng T, Hu X and Li X: MicroRNA-423 drug resistance and proliferation of breast cancer cells by targeting ZFP36. Onco Targets Ther 13: 769-782, 2020].

Owing to the fact that the contentious data in the above article had already been submitted for publication prior to its submission to *Experimental and Therapeutic Medicine,* the Editor has decided that this paper should be retracted from the Journal. The authors were asked for an explanation to account for these concerns, but the Editorial Office did not receive a reply. The Editor apologizes to the readership for any inconvenience caused.

